# Supplementary material for: The causal relationship between the human gut microbiota and pyogenic arthritis: a Mendelian randomization study
Source: Front Cell Infect Microbiol. 2024 Nov 26;14:1452480. doi: 10.3389/fcimb.2024.1452480 (PMC11629706; doi:10.3389/fcimb.2024.1452480)
Supplement: Supplementary file 1 [file DataSheet1.zip › mendelian test/ebi-a-GCST90027610.csv_leave_one_out.pdf]

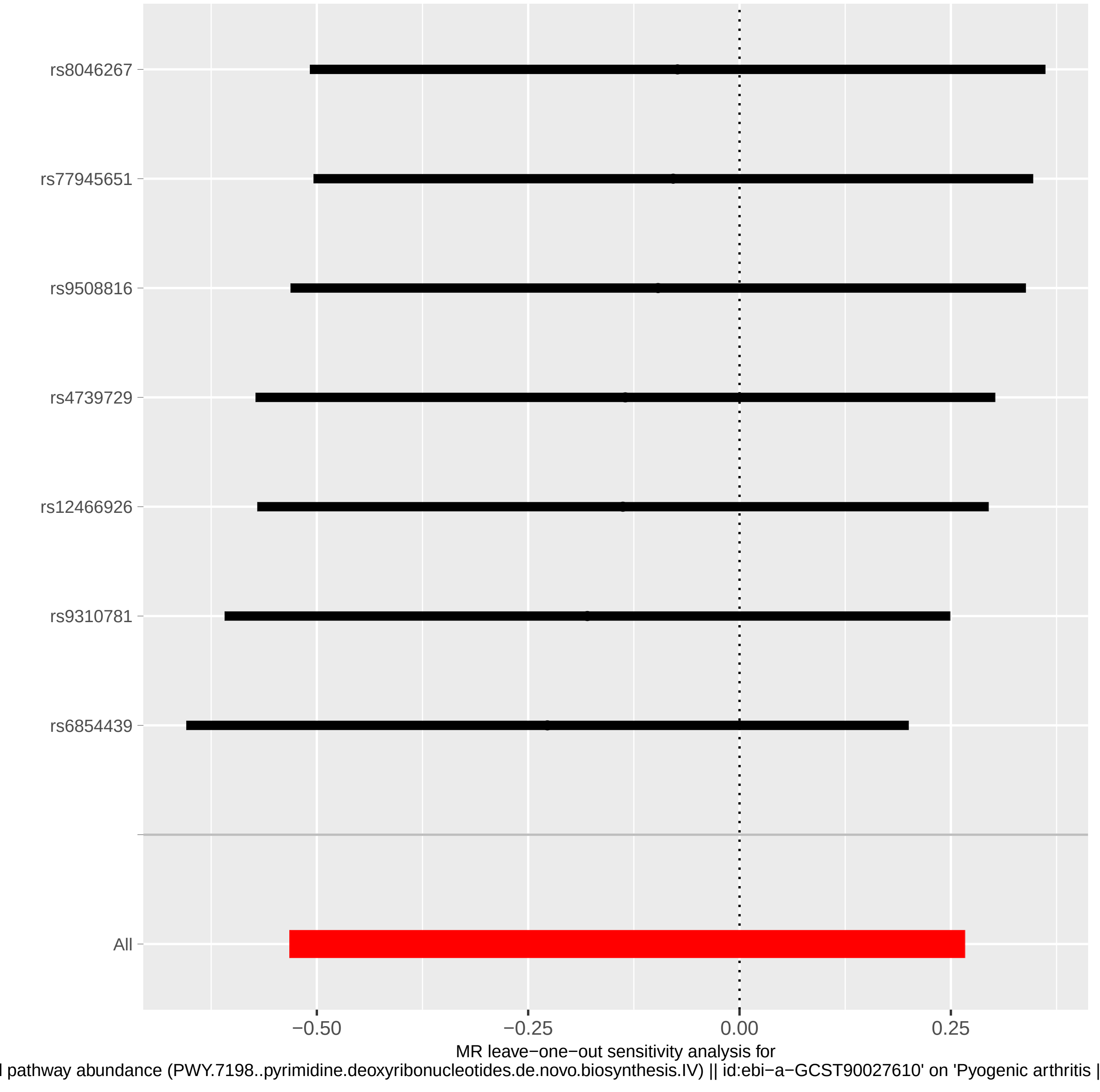
rs8046267
rs77945651
rs9508816
rs4739729
rs12466926
rs9310781
rs6854439
All
−0.50
−0.25
0.00
0.25
MR leave−one−out sensitivity analysis for
l pathway abundance (PWY.7198..pyrimidine.deoxyribonucleotides.de.novo.biosynthesis.IV) || id:ebi−a−GCST90027610' on 'Pyogenic arthritis |
